# Selective Sorption of Dissolved Organic Carbon Compounds by Temperate Soils

**DOI:** 10.1371/journal.pone.0050434

**Published:** 2012-11-27

**Authors:** Sindhu Jagadamma, Melanie A. Mayes, Jana R. Phillips

**Affiliations:** Climate Change Science Institute and Environmental Sciences Division, Oak Ridge National Laboratory, Oak Ridge, Tennessee, United States of America; National University of Singapore, Singapore

## Abstract

**Background:**

Physico-chemical sorption onto soil minerals is one of the major processes of dissolved organic carbon (OC) stabilization in deeper soils. The interaction of DOC on soil solids is related to the reactivity of soil minerals, the chemistry of sorbate functional groups, and the stability of sorbate to microbial degradation. This study was conducted to examine the sorption of diverse OC compounds (D-glucose, L-alanine, oxalic acid, salicylic acid, and sinapyl alcohol) on temperate climate soil orders (Mollisols, Ultisols and Alfisols).

**Methodology:**

Equilibrium batch experiments were conducted using 0–100 mg C L^−1^ at a solid-solution ratio of 1∶60 for 48 hrs on natural soils and on soils sterilized by γ-irradiation. The maximum sorption capacity, *Q_max_* and binding coefficient, *k* were calculated by fitting to the Langmuir model.

**Results:**

Ultisols appeared to sorb more glucose, alanine, and salicylic acid than did Alfisols or Mollisols and the isotherms followed a non-linear pattern (higher *k*). Sterile experiments revealed that glucose and alanine were both readily degraded and/or incorporated into microbial biomass because the observed *Q_max_* under sterile conditions decreased by 22–46% for glucose and 17–77% for alanine as compared to non-sterile conditions. Mollisols, in contrast, more readily reacted with oxalic acid (*Q_max_* of 886 mg kg^−1^) and sinapyl alcohol (*Q_max_* of 2031 mg kg^−1^), and no degradation was observed. The reactivity of Alfisols to DOC was intermediate to that of Ultisols and Mollisols, and degradation followed similar patterns as for Ultisols.

**Conclusion:**

This study demonstrated that three common temperate soil orders experienced differential sorption and degradation of simple OC compounds, indicating that sorbate chemistry plays a significant role in the sorptive stabilization of DOC.

## Introduction

Organic carbon (OC) in soil is the largest C sink in the biosphere and storing this pool is important to combat climate change. One third of the 2344 Pg C stored in soils is located in profiles below 1 m [1]. The C stored in deeper soil horizons is characterized by decreased C:N ratio and increased ^14^C age, indicating that subsoil C is more stabilized than the surface soil C [2]–[3]. The longer stability of OC in subsoil than surface soil is attributed to several reasons including more favorable physico-chemical properties of subsoil minerals for sorption [4], [5], non-availability of fresh OC in subsoil to provide sufficient energy to microbial sustenance [6] and reduced microbial activities due to sub-optimal environmental conditions [7]. As a consequence of one or more of above reasons, deeper horizons of mature soils such as Alfisols, Ultisols and Oxisols possess a potential of storing 165 Pg C for each meter of soil [8], [9]. Thus, subsoil C storage has a significant role in determining the extent to which soils provide a sink for atmospheric CO_2_.

Sorption of dissolved organic carbon (DOC) on soil minerals is considered to be the major process of OC preservation in deeper soils, accounting for 25–98% of the measured soil OC (SOC) stock [10]. Currie et al. [11] reported that 217–366 kg C ha^−1^ yr^−1^ is retained in the mineral forest subsoil by sorption. The retention of DOC is influenced by the chemical properties of mineral sorption sites [12] and in general, the primary sorbents for DOC are minerals dominated by variable charges [13]. Close correlations between Fe and Al oxyhydroxides and OC concentrations in soils were reported in many studies [14]–[16], and strong binding of OC functional groups on Fe and Al oxide surfaces limits desorption [17], [18]. Kothawala et al. [19] found that poorly crystalline Fe and Al minerals contributed more than the total clay content to the sorption capacity of a wide range of soils. They did not find any influence of soil OC content to sorption capacity of soils. A recent study using 213 subsoils from US provided evidence that bulk DOC sorption was correlated with different properties for Mollisols, Ultisols and Alfisols [20]. When the soils were grouped, sorption capacity correlated with clay and Fe content. However, when data was analyzed separately for soil orders, clay was most important for Alfisols and Ultisols, and total OC content was most important for Mollisols. Cecchi et al. [21] studied the sorption-desorption characteristics of several phenolic acids in a wide array of US soils and reported that phenolic acid sorption was more strongly correlated to clay content than pH and OC, and all the three properties together predicted the sorption of most phenolic acids better than individual properties.

**Table 1:** General characteristics of soils.

Soil order	Soil series	Location	Taxonomy	Land use	Clay	Fe	pH	TOC	TIC
					(%)	(mg g^−1^)^a^		(mg kg^−1^)^b^	(mg kg^−1^)^c^
Mollisols	Drummer	Kane, IL	Endoaquolls	Grassland	18	18	6.5	2697	32362
	Longford	Washington, KS	Argiustolls	Grassland	36	6.7	6.6	2902	553
	Pawnee	Lancaster, NE	Argiustolls	Grassland	30	16	7.7	1811	19620
Ultisols	Jefferson	Anderson, TN	Hapludults	Woodland	42	22	6.3	1956	613
	Collegedale	Anderson, TN	Paleudults	Grassland	55	29	5.9	2985	1229
	Wolftever	Anderson, TN	Hapludults	Cropland	30	15	4.4	2309	640
Alfisols	Malmo	Lancaster, NE	Hapludalfs	Grassland	37	11	6	3240	421
	Arispe	Decatur, IA	Hapludalfs	Cropland	51	11	6.6	1961	149
	Zanesville	Spencer, IN	Fragiudalfs	Cropland	40	26	5.5	1856	143

a Total iron oxide by dithionite-citrate-bicarbonate method. Data adapted from Mayes et al. [20].

b Total organic carbon.

c Total inorganic carbon.

Chemistry of sorbate molecules also has a profound influence in controlling the extent and stability of DOC sorption. In general, hydrophobic OC compounds appear to exhibit preferential sorption over hydrophilic compounds due to multitude of reasons such as high molecular weight, favorable interactions of functional groups with the sorbent surfaces, favorable steric arrangement of functional groups, and greater proportion of *o*-hydroxy-benzoic acid structures which favors ligand exchange mechanism of sorption [17], [22]–[25]. It is also generalized that sorption of high molecular weight macromolecules on soil minerals are mostly irreversible due to the formation of highly stable organo-mineral polydentate surface complexes [26]. Mayes et al. [20] found significant differences in the Langmuir binding coefficient (*k*) depending on the soil order, where the *k* of Mollisols was much lower than that of Ultisols. A low *k* indicates a relatively flat isotherm requiring substantial addition of DOC to reach the maximum sorption capacity, and therefore *k* is an indication of selectivity for the DOC sorbate. As discussed above, selectivity can be related to the chemical structure of functional groups present in heterogeneous natural DOC, as well as the difference in reactivity of the minerals. Van Hees et al. [27] examined the sorption of three organic acids (acetate, oxalate and citrate) across three forest soil types, and found that maximum sorption capacity and the shape of the isotherm varied widely depending on the organic acid sorbent, following the order oxalate≥citrate>>acetate. Sorption isotherms of 16 chemically diverse compounds on Pahokee peat soil demonstrated that the concentration and polarizability of the sorbates were responsible for the strength and non-linearity of sorption [28]. Thus, solution chemistry, as well as mineral composition, can influence the extent of sorption.

**Figure 1:**
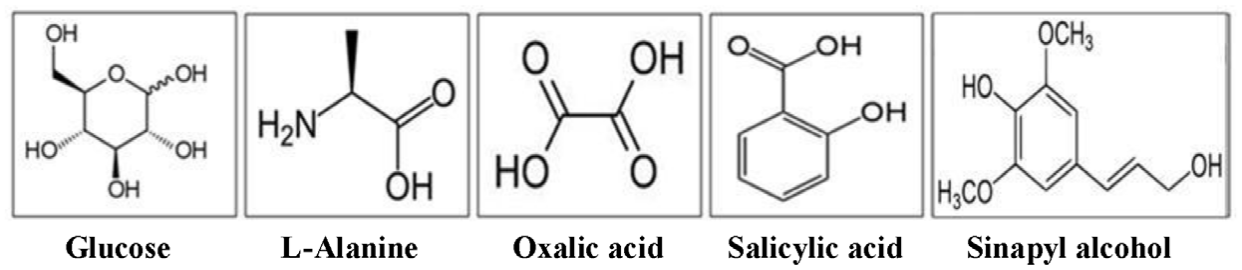
Chemical structures of selected organic compounds.

Studies that investigated the sorption of specific compounds, usually low molecular weight organic acids, observed significant biodegradation of the sorbates during the sorption experiments [29]–[32]. Therefore OC incorporation into microbial biomass and/or mineralization through microbial respiration can be erroneously attributed to sorption [33]–[35]. Some sorption studies assured absence of microbial activities by sterilizing the soils before the sorption experiments [27], [31], [36]. Fischer et al. [36] demonstrated that sorption of glucose, alanine and acetate under sterile conditions were 26%, 27% and 21% of the apparent sorption observed when experiments were conducted under non-sterile conditions. Thus, microbial and sorptive processes need to be segregated for accurate quantification of the soil capacity for adsorption of DOC compounds.

**Table 2:** Maximum sorption capacity (*Q_max_*) and binding coefficient (*k*) determined by fitting to the Langmuir model.

Compounds	Soil order	*Q_max_* (mg kg^−1^)	*k* (L mg^−1^)
D-Glucose	Mollisols	35 (2.8)^b^	0.024 (0.009)^b^
	Ultisols	173 (14.3)^a^	0.141 (0.01)^a^
	Alfisols	126 (9.8)^ab^	0.067 (0.01)^b^
L-Alanine	Mollisols	98 (7.9)^b^	0.037 (0.002)^b^
	Ultisols	527 (48.1)^a^	0.193 (0.02)^a^
	Alfisols	444 (52.4)^a^	0.059 (0.01)^b^
Salicylic acid	Mollisols	240 (43.7)^b^	0.055 (0.01)^ab^
	Ultisols	502 (70.4)^a^	0.103 (0.03)^a^
	Alfisols	481 (98.9)^a^	0.032 (0.01)^b^
Sinapyl alcohol	Mollisols	2031(182)^a^	0.008 (0.003)
	Ultisols	214 (16.2)^c^	0.009 (0.002)
	Alfisols	521 (53.9)^b^	0.006 (0.002)
Oxalic acid	Mollisols^*^	886 (100)^a^	0.101 (0.01)b
	Ultisols	471 (28.5)^b^	0.164 (0.01)a
	Alfisols	955 (122)^a^	0.105 (0.01)b

Results are shown as mean (standard error). *Q_max_* and *k* values followed by different lowercase letters within a compound is statistically significant at *P≤*0.05. ^*^ Represent only one soil series (Longford). In all other cases, data from three soils series were averaged for each soil order.

It is clear that sorptive stabilization of OC on soils is determined by the combined influence of sorbent and sorbate properties, as well as microbial bioavailability. In this study, we used three soil orders (Ultisol, Mollisol and Alfisol) which are dominant in the U.S. Ultisols are highly weathered and dominated by Fe and Al oxide minerals, while the Mollisols are least weathered and dominated by 2∶1 and 2∶2 layered silicate clays [37]. The moderate reactivity of Alfisols is in alignment with its intermediate maturity between Ultisols and Mollisols [38]. Positively charged Fe and Al oxide minerals such as common to Ultisols are expected to exhibit stronger affinity to a wider range of OC functional groups as compared to negatively charged clay minerals in Mollisols [8]. Based on this understanding of soil mineralogy and following Mayes et al. [20], we hypothesized that Ultisols would exhibit little preference for various functional groups of DOC, while Mollisols would preferentially sorb only specific functional groups of natural DOC. Thus the present study was conducted with the following specific objectives: (i) compare the sorption capacity of three soil orders representative of temperate climates (Mollisols, Ultisols and Alfisols) upon the addition of the selected OC compounds (D-glucose, L-alanine, salicylic acid, sinapyl alcohol and oxalic acid), (ii) determine the physico-chemical properties of soils most related to sorption capacity of different compounds, and (iii) quantify the biodegradation of the added compounds by comparing the sorption capacity of sterile and non-sterile soils.

**Figure 2:**
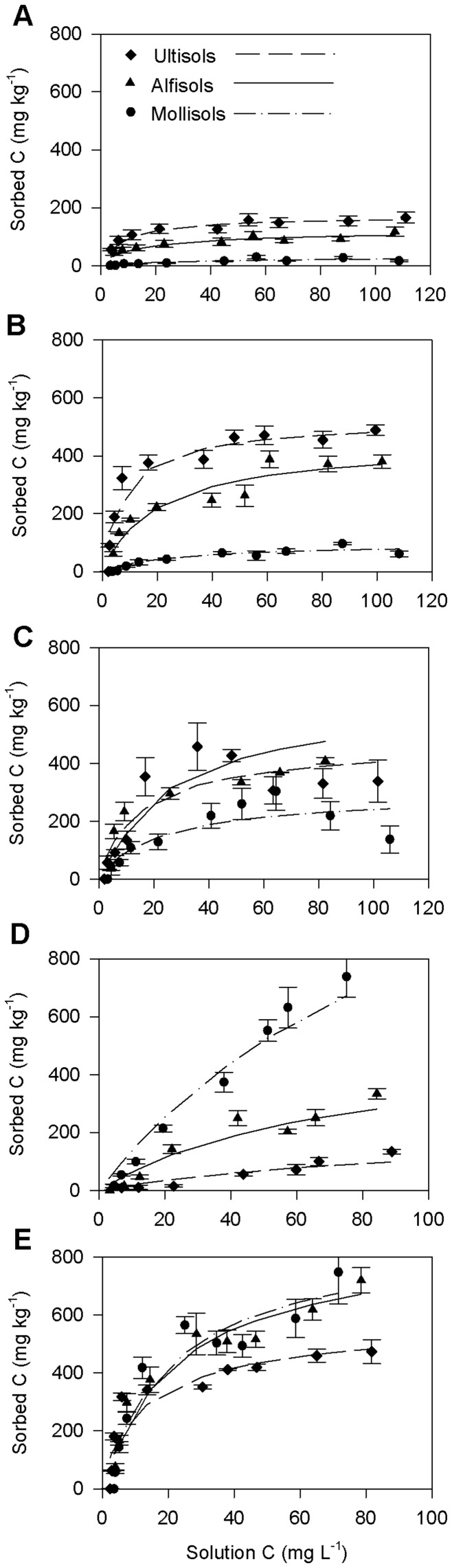
Sorption isotherms and Langmuir fits of organic carbon compounds on different soil orders. (A) D-Glucose, (B) L-Alanine, (C) Salicylic acid, (D) Sinapyl alcohol and (E) Oxalic acid. Each isotherm per soil order is an average of three soils except for Mollisols in (E) where only one soil type (Longford) is considered. Symbols correspond to measured sorption and lines correspond to Langmuir model predicted sorption.

**Table 3:** Linear regression coefficients (R^2^) between maximum sorption capacity (*Q_max_*) and soil properties.

Compounds	Clay	pH	TOC	Fe	All four variables
D-Glucose	NS^a^	NS	NS	NS	NS
L-Alanine	0.51	NS	NS	NS	0.78
Salicylic acid	0.76	NS	NS	0.49	0.82
Sinapyl alcohol	0.62	0.42	NS	0.53	0.75
Oxalic acid	0.67	0.47	NS	0.61	0.82

a Not significant at *P≤*0.05.

## Materials and Methods

### Soils

Subsoil samples (B horizon) from nine soils representing Mollisols, Ultisols and Alfisols were selected for this study. The selected soil series are Drummer, Longford and Pawnee for Mollisols, Jefferson, Collegedale and Wolftever for Ultisols, and Malmo, Arispee and Zanesville for Alfisols. These soils were carefully selected from a soil database of 213 temperate subsoils [20] with the criteria that the sorption parameters of individual soils when treated with a bulk DOC sorbate should be closer to the average of their soil order. Accordingly, the sorption parameters (maximum sorption capacity, *Q_max_* and binding coefficient, *k*) and major physico-chemical properties (clay, soil pH, total OC and total Fe oxides) of the selected soils mostly fell within the 25^th^ and 75^th^ quartile of 49 Mollisols, 59 Ultisols and 86 Alfisols soils used in Mayes et al. [20]. Therefore the 3 soils chosen from each order may be considered to be broadly representative of the corresponding soil order. Samples were air dried and sieved through a 2 mm sieve. Basic physical and chemical properties of the <2 mm soils (clay content, total Fe oxides, soil pH, total organic and inorganic carbon) were reported in Mayes et al. [20] and a summary is provided in Table 1. Clay content was determined from the soil textural analysis using the bouycous hydrometer method [39]. Total Fe oxides were extracted from the soils with the dithionate-citrate-bicarbonate extractant [40] followed by analysis using inductively coupled plasma mass spectrometry (ELAN-6100, PerkinElmer Corp., Waltham, MA). Soil pH was determined by shaking the soil in 5 mM CaCl_2_ (1∶2 soil to solution ratio) and measuring the pH of the supernatant using a pH meter [41]. Total C and organic carbon were measured by combustion method using a CN analyzer (LECO Corp., St. Joseph, MI) [42]. Before the analysis, the sub-samples were treated with 3 M HCl for 1 hr in order to remove the inorganic carbon. Results from the untreated samples correspond to the total C and that from the HCl treated samples correspond to organic carbon. Inorganic carbon was computed from the difference of untreated and treated soils.

**Figure 3:**
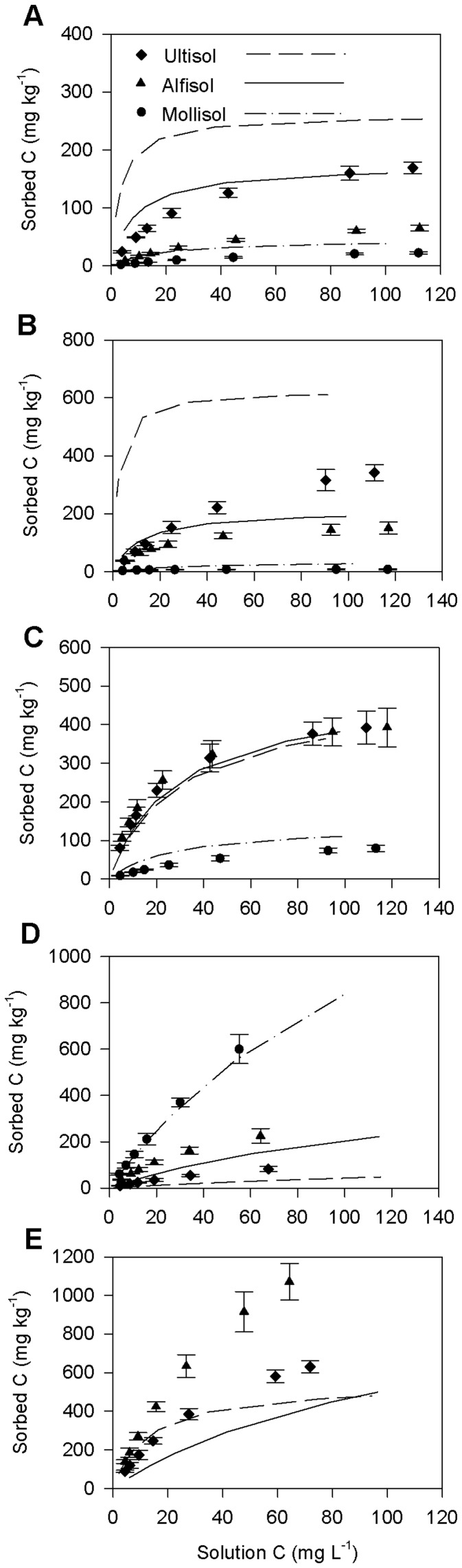
Sorption comparison between sterile and non-sterile Drummer, Jefferson and Malmo soils. (A) D-Glucose, (B) L-Alanine, (C) Salicylic acid, (D) Sinapyl alcohol and (E) Oxalic acid. Symbols correspond to sorption of sterile soils and lines correspond to sorption of non-sterile soils. For (E), data from Drummer soil was not considered because precipitation reaction was predominant than sorption.

**Table 4:** Comparison of Langmuir sorption parameters of sterile soils with corresponding non-sterile soils.

		Q_max_ (mg kg^−1^)		*k* (L mg^−1^)	
Compounds	Soil series (order)	Non-Sterile	Sterile	Non- sterile	Sterile
D-Glucose	Drummer (Mollisol)	45 (2.6) ^a1^	35 (1.8)^b^	0.054 (0.004)^a^	0.016 (0.003)^b^
	Jefferson (Ultisols)	261 (30.4)^a^	189 (15.1)^b^	0.294 (0.08)^a^	0.033 (0.001)^b^
	Malmo (Alfisols)	173 (16.1)^a^	94 (3.9)^b^	0.116 (0.03)^a^	0.02 (0.001)^b^
L-Alanine	Drummer (Mollisol)	35 (2.9)^a^	8 (0.2)^b^	0.03 (0.008)	0.01 (0.001)
	Jefferson (Ultisols)	626 (50.5)^a^	435 (23.3)^b^	0.45 (0.03)^a^	0.02 (0.001)^b^
	Malmo (Alfisols)	213 (30.4)	176 (12.6)	0.09 (0.01)	0.05 (0.001)
Salicylic acid	Drummer (Mollisol)	140 (22.0)	121 (9.2)	0.04 (0.01)	0.02 (0.001)
	Jefferson (Ultisols)	544 (71.9)	567 (36.7)	0.08 (0.008)	0.05 (0.001)
	Malmo (Alfisols)	521 (39.1)	549 (25.0)	0.04 (0.003)	0.06 (0.001)
Sinapyl alcohol	Drummer (Mollisol)	2107 (330)	2350 (266)	0.007 (0.001)	0.006 (0.002)
	Jefferson (Ultisols)	150 (9.3)	186 (16.9)	0.004 (0.001)	0.007 (0.001)
	Malmo (Alfisols)	500 (29.9)	607 (49.1)	0.007 (0.001)	0.01 (0.008)
Oxalic acid	Drummer (Mollisol)	ND	ND	ND	ND
	Jefferson (Ultisols)	383 (50.3)^b^	825 (54.8)^a^	0.11 (0.004)	0.09 (0.002)
	Malmo (Alfisols)	1061 (221)^b^	2107 (139)^a^	0.01 (0.008	0.02 (0.001)

Results are shown as mean (standard error).

*Q_max_* and *k* values followed by different letters in parenthesis between non-sterile and sterile soils within a compound and soil order is statistically significant at *P≤*0.05. Only one soil type per order is compared, so the *Q_max_* and *k* of non-sterile soils do not match with that in Table 2.

NA = Not determined.

### Soil Sterilization

Selected soils (Drummer, Jefferson and Malmo) were sterilized by γ-irradiation by placing sub-samples into polypropylene tubes and γ-irradiated by A.J.L. Shepherd ^60^Co irradiator (model # 7810-0109-R) at the Oak Ridge National Laboratory. Published literature demonstrates that γ-irradiation is superior to addition of toxic chemicals (e.g. Na-azide, HgCl_2_) or autoclaving in suppressing microbial activity and in minimizing changes in soil properties [33], [43]–. Samples were sterilized for 84 hours at a rate of 3.92 Gy min^−1^ with a resultant γ-ray dose of 20 kGy. Bank et al. [47] conducted γ-irradiation of similar soils using similar γ-ray dose and detected no viable microbial cells in the sterilized soils. They also reported that the major soil properties and clay mineralogy were unchanged after sterilization. The soils were stored in closed vials in a sterile environment until the beginning of the sorption experiments.

### Organic Carbon Compounds

Five synthetic OC compounds were used in this study as sorbates. The selected sorbates represent diverse classes of dissolved OC compounds commonly present in SOC. We selected synthetic compounds to mimic the chemistry of natural SOC based on abundance in soil, representation of both open chain and ring structures, and water solubility. The selected compounds are D-glucose (carbohydrates), L-alanine (amino acids), salicylic acid (phenolic compound), sinapyl alcohol (lignin monomer) and oxalic acid (carboxylic acid) (Fig. 1). Ten concentrations (0, 2, 5, 10, 20, 40, 50, 60, 80, 100 mg C L^−1^) of initial solutions of these compounds were prepared. Background solution used was 0.01 M NaCl in MQ water. Fresh solutions were prepared in ashed glassware immediately before the start of each sorption experiment to limit the opportunity for microbial inoculation of the solutions or the soils.

### Sorption Experiments

Batch sorption experiments were performed in triplicate in 40 mL ashed glass centrifuge tubes at a constant ionic strength of 0.01 using NaCl, indigenous soil pH, and room temperature [8], [17], [20], [22]. Thirty mL of DOC solutions from each concentration ranging from 0 to 100 mg C L^−1^ was added to glass vials containing 0.5 g of each soil. After shaking gently on a reciprocal shaker for 48 hours, the mixtures were centrifuged at 3000 rpm for 10–15 minutes and the supernatants were collected. Changes in pH before and after reaction with soils was monitored and found out no significant difference in pH before and after the sorption experiments. Another set of batch experiments were conducted with γ-irradiated soils (Drummer, Jefferson and Malmo) aseptically by performing every aspect of the experiment in a sterile hood except that the soil solutions were shaken and centrifuged outside the fume hood, but the vials were closed. Supernatants from both non-sterile and sterile sorption experiments were analyzed for total dissolved OC (TOC) using Shimadzu Total Organic Carbon Analyzer (TOC-5000). Blanks were analyzed in triplicate for each soil to determine the amount of TOC desorbed from the indigenous SOC. Recovery of the added OC compounds in the equilibrium solution phase after the sorption experiment were detected by analyzing selected samples (with initial concentration of 20, 40 and 80 mg C L^−1^) using high performance liquid chromatography (HPLC). We detected D-glucose using a Flexar HPLC system (Perkin Elmer) and L-alanine, oxalic acid and salicylic acid using Ultimate 3000 system (Dionex). We were unable to detect sinapyl alcohol using either of these systems. The Flexar system used Biorad HPX-87P with guard column and Refractive Index detector for D-glucose detection with HPLC grade water as the mobile-phase. The Ultimate 3000 system used Acclaim Mixed-Mode Wax-1 column and Ultraviolet detector for the detection of other compounds with different proportions of acetonitrile and phosphate buffer as the mobile phase. The compound recovery data from HPLC analysis was normalized to C concentrations in order to make comparisons with TOC results and we found good agreement between HPLC and TOC results.

### Isotherm Modeling

Sorption isotherms generated from the experimental data were fitted using the Langmuir equation [17], [48],
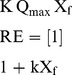
where RE is amount of DOC adsorbed or desorbed (mg kg^−1^ soil), *X_f_* is equilibrium final solution concentration (mg L^−1^), *Q_max_* is maximum sorption capacity (mg kg^−1^), and *k* is binding coefficient (L mg^−1^). Potential desorption ‘*b*’, which is an estimate of the amount of indigenous TOC desorbed from the soil was calculated from the average of triplicate “blanks” involving addition of 0 mg C L^−1^ to the soil [19]. Eq. [1] is modified by including the term ‘*b*’ which allowed an adjustable y intercept. The modified Langmuir equation is represented as,



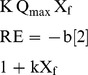
RE in Eq. [2] represents the additional TOC adsorbed and *Q_max_* represents the maximum additional OC sorption capacity [19], [20]. The *Q_max_* and *k* were determined by fitting the experimental data for each of the three replicate isotherms using a non-linearized optimizer [49].

### Statistical Analysis

The analysis of variance (ANOVA) for testing the difference among soil orders for *Q_max_* and *k* for each compound was computed using PROC GLM procedure (fixed effects model) of SAS [50]. Statistical significance was evaluated at *P≤*0.05 level and the mean effects were separated using the F protected least significant difference (LSD) test. The standard error of estimates was not considered for ANOVA because we noted that the sorption parameters do not overlap across the soil orders even after considering the standard error of estimates. Since the sorption parameters were statistically similar within a soil order, the *Q_max_* and *k* values reported in Table 2 were obtained by averaging 9 observations (3 soil types × 3 analytical replications).

To determine correlations between mineral properties and observed sorption for all three soil orders together, linear regressions were performed between *Q_max_* and major soil properties using PROC REG option of SAS. The regression model was considered statistically significant when *P≤*0.05. The major soil properties were considered individually and together. Note that we did not perform separate regression analysis for each soil order because the sample size within a soil order (n = 3) was insufficient, and the standard error associated with *Q_max_* and *k* estimation were also not considered.

## Results and Discussion

The overall goal of this study was to test the hypothesis that sorption behavior of different soil orders is influenced by the chemistry of sorbate compounds and that different soil orders show affinity to different classes of OC compounds. In general, Ultisol and Alfisol soils contained more clay than Mollisol soils (Table 1). The pH of Mollisols were in the upper range (6.5–7.7), but the pH of Ultisols varied in a broader range (4.4–6.3). Two out of three Mollisols exhibited very high total inorganic carbon content which is typical of Mollisol sub soils. Total Fe content was higher for Ultisol as compared to other soils. We found that sorption parameters (*Q_max_* and *k*) of individual soils within each soil order, as calculated from isotherm modeling, are statistically similar (*P*>0.05) to each other, except for oxalic acid sorption on Mollisols (*F*_2,6_ = 9.4, *P* = 0.02). Therefore sorption parameters of individual soils within each order were combined together to derive the average sorption parameters of particular soil order (Table 2).

### Sorption Parameters

Among the compounds tested, D-glucose showed the least reactivity to the solid phase (Fig. 2). Maximum sorption capacity *Q_max_* with glucose addition was the lowest for Mollisols (35 mg kg^−1^) and highest for Ultisols (173 mg kg^−1^) (F_2,6_ = 5.7, *P* = 0.04) (Table 2). *Q_max_* of Alfisols for D-glucose (126 mg kg^−1^) was intermediate to that of Mollisols and Ultisols. The binding coefficient *k* also showed significant differences across soil orders, with statistically higher values for Ultisols than Alfisols and Mollisols (F_2,6_ = 7.9, *P* = 0.03) (Table 2). Poor sorption of glucose to soil minerals was consistent with previous results, Kuzyakov and Jones [51] determined that over 90% of added glucose remained in solution phase after 1 hr in sterile soils, while Jones and Edwards [52] attributed poor sorption of glucose to its lack of charge. Some past studies suggested that glucose sorption to mineral surfaces was primarily through hydrogen bonding and partly through hydrophobic interactions [53], [54]. With the help of spectroscopic techniques, Liu et al. [55] and Olsson et al. [56] confirmed that the predominant mechanism of interaction of glucose with mineral surfaces is hydrogen bonding. Nonetheless, our results did not indicate evidence for a strong interaction of glucose with soil minerals. In addition, no statistically valid relationships were established from simple linear regressions between *Q_max_* and common soil properties (clay content, pH, TOC and Fe content) and from multiple regression including all the properties together (Table 3) confirming lack of specific binding mechanisms of glucose with soil minerals in our study.

L-alanine was chosen as the model amino acid because of its high relative abundance in bulk DOC [34], [57], [58]. Across different soil orders, L-alanine sorption followed a pattern similar to that of D-glucose with Mollisols registering the least sorption (*Q_max_* 98 mg kg^−1^) followed by Alfisols (*Q_max_* 444 mg kg^−1^) and Ultisols (*Q_max_* 527 mg kg^−1^) (F_2,6_ = 9.2, *P* = 0.02) (Table 2). The binding coefficient *k* and the isotherm shape were similar to that of glucose (F_2,6_ = 7.4, *P* = 0.03) (Fig. 2, Table 2). Comparison of *Q_max_* values revealed that L-alanine was more reactive than D-glucose, especially to Ultisols and Alfisols. A significant linear regression relationship observed between clay content and *Q_max_* for L-alanine sorption suggests that more clayey Ultisols and Alfisols are more reactive to L-alanine than less clayey Mollisols (Table 3).

The sorption capacity of the model aromatic acid, salicylic acid, was also higher for Ultisols (502 mg kg^−1^) and Alfisols (481 mg kg^−1^) than Mollisols (240 mg kg^−1^) (F_2,6_ = 6.9, *P* = 0.03). The binding coefficients and isotherm curves were similar to that of glucose and alanine sorption (Fig. 2, Table 2) (F_2,6_ = 8.9, *P* = 0.02). The linear regression results indicated that clay and Fe oxide contents explain most of the variations of salicylic acid sorption across the three soil orders (Table 3). Celis et al. [59] reported that soils with more positive charged soil components such as Fe oxides played a significant role in the sorption of salicylic acid. It is also possible that this aromatic acid forms bidentate complexes with sesquioxides and positively charged metal oxides [60]. In our study, average Fe oxide content was highest for Ultisols (22 mg g^−1^) as compared to Alfisols (16 mg g^−1^) and Mollisols (13.6 mg g^−1^), which suggests that sorption to positively-charged soil components could account for the observed sorption of salicylic acid.

Sinapyl alcohol sorption followed more of a linear trend for all the soils tested as evidenced by the shape of the isotherms (Fig. 2) and also from the lower and statistically similar *k* values (Table 2). However, the *Q_max_* values showed strong statistical differences across soil orders: *Q_max_* was significantly higher for Mollisols (2031 mg kg^−1^) than Alfisols (521 mg kg^−1^) and Ultisols (214 mg kg^−1^) (F_2,6_ = 149, *P* = <0.0001). This is in contrast to the response of glucose, alanine and salicylic acid. Cecchi et al. [21] also observed a rapid sorption of lignin derivatives similar to sinapyl alcohol on several US Mollisols. Higher sinapic acid sorption was also reported by Lehmann and Cheng [61] in three eastern Washington grasslands (Mollisols) when compared to eight western Washington forest soils (mostly Inceptisols). Due to its complex structure, the exact mechanisms of interaction of lignin molecules with soil minerals have not yet been elucidated [62]. Two possibilities are that lignin derivatives could be bonded irreversibly onto organometallic compounds by polymerization reactions, or covalently to mineral or organic particle surfaces [21], [63]. The polymerization appears to occur mostly in soils dominated by 2∶1 and 2∶2 minerals [64]. Thus, higher sorption of sinapyl alcohol on Mollisols could be linked to the dominance of 2∶1 minerals in these soils and/or to binding to soil OC coating on Mollisols minerals as suggested by the correlations between OC and sorption of natural DOC on Mollisols [20]. Cecchi et al. [21] also reported significant relationships between soil OC content and sorption of compounds structurally similar to sinapyl alcohol. Soil organic carbon content did not show statistically valid relationship with *Q_max_* of any of the tested compounds (Table 3), which could be due to the narrow range of OC values of our tested soils (Table 1).

Two out of the three Mollisols studied (Drummer and Pawnee) showed high sorption of oxalic acid: *Q_max_* of Drummer (5400 mg kg^−1^) and Pawnee (5180 mg kg^−1^) were one order of magnitude higher than that of Longford (886 mg kg^−1^). Oxalate reacts with free Ca^2+^ in soil solution and precipitates as insoluble Ca oxalate [23], [31], [55]. Ström et al. [65] reported a five-fold increase in apparent sorption of oxalate than malate and citrate on calcareous soils. Both Drummer and Pawnee are calcareous soils as indicated by their total inorganic carbon content (Table 1). Thus, the sorption calculation for oxalic acid in calcareous soils most likely includes precipitation reactions. Isotherm and sorption parameters for Mollisols presented in Fig. 2 and Table 2 corresponds to the data from only non-calcareous Mollisols (Longford) (F_2,6_ = 7.6, *P* = 0.03). Data from three analytical replicates of Longford is used as such in this case for valid ANOVA test. Both Longford and Alfisols showed statistically higher *Q_max_* than Ultisols for oxalic acid. However, the *Q_max_* of Ultisols (471 mg kg^−1^) was comparable to the average *Q_max_* values exhibited by Ultisols with the sorption of other compounds (173–527 mg kg^−1^) (Table 2). In general, sorption of carboxylic acids on soil solids is thought to be a quick process with more than 70% of the sorption occurring within 1 min [66]–[68]. The relatively higher *Q_max_* and *k* values as a result of oxalic acid sorption in our study were also indicative of the fact that soil minerals possess a strong affinity to oxalic acids (Table 2). All the soil properties except OC content demonstrated influence on explaining the variations in oxalic acid sorptivity of soils (Table 3).

Different soil orders exhibited differential sorptive responses to diverse classes of OC compounds. Ultisols exhibited more sorption of glucose, alanine and salicylic acid than Alfisols and Mollisols. On the other hand, Mollisols and Alfisols were superior to Ultisols in terms of oxalic acid and sinapyl alcohol sorption. The binding coefficient *k* was significantly higher for Ultisols upon the addition of most of the compounds which implies that Ultisols responded more non-linearly to these compounds with strong initial sorption followed by an indication of saturation of sorption sites with higher concentration of compounds (Fig. 2). This is in agreement with Mayes et al. [20] reporting higher average *k* values for Ultisols (0.09 L mg^−1^) than Alfisols (0.05 L mg^−1^) and Mollisols (0.02 L mg^−1^) as a result of the sorption with a natural bulk DOC solution. As explained in Mayes et al. [20], more non-linear isotherms for Ultisols demonstrates that at low concentrations, the added compounds were readily sorbed onto high affinity sites and the maximum sorption was achieved with relatively low concentration of added compounds. Irrespective of the type of compounds added, here *Q_max_* of Ultisols varied within a narrow range of 173 to 527 mg kg^−1^ (Table 2). This suggests that Ultisols were not very selective for the type of added compound. In contrast, lower *k* values for Mollisols imply that these soils typically possess fewer high affinity sites and these sites respond mostly to particular OC compounds such as sinapyl alcohol and oxalates. Consequently, observed greater selectivity for the type of compound added resulted in wide variation of *Q_max_* for Mollisols (35–2031 mg kg^−1^). The *Q_max_* of Alfisols were intermediate between Mollisols and Ultisols.

### Sorption of Sterile and Non-sterile Soils

Because rapid microbial uptake is possible for simple organic compounds such as glucose, we determined the extent of microbial degradation for selected soils from the sorption study by comparing the *Q_max_* of replicate samples of sterile and non-sterile soils. Since sorption characteristics of all the non-sterile soils within each soil order showed statistical similarity, we sterilized only one soil per order (Drummer, Jefferson and Malmo). Consistently higher apparent sorption was observed for D-glucose and L-alanine with non-sterile soils than with the sterilized soils (Fig. 3a,b; Table 4) and the F_1,4_ statistic across the soils ranged from 9.2–12.4 for D-glucose sorption (*P* = 0.008–0.03) and 8.7–10.1 for L-alanine sorption (*P* = 0.02–0.04). Note that isotherm parameters in Table 4 are from one soil series per order (Drummer, Jefferson and Malmo) while those in Table 2 are an average of three soil series per order. Higher apparent sorption in D-glucose and L-alanine in the non-sterile experiments as compared to sterile experiments clearly demonstrates utilization of substrates by the microbial community in the former. Under sterile conditions, *Q_max_* decreased by 22–46% for glucose and 17–77% for alanine (Table 4). The *Q_max_* of Ultisol (Jefferson) of D-glucose and L-alanine remained the highest of the three orders under both non-sterile and sterile conditions. This confirms that among the soil orders studied, Ultisols exhibited the highest affinity to both D-glucose and L-alanine, despite apparent microbial uptake. Note that our experiments can only determine that the microbial community incorporated the substrate and we could not determine if the substrate was respired.

There was no indication of substantial microbial uptake when salicylic acid and sinapyl alcohol were added to soils, as evidenced by statistically similar sorption parameters under sterile and non-sterile conditions (Fig. 3c,d,; Table 4). Biodegradation of ring structured compounds is thought to be a slow process due to the greater thermodynamic stability of the ring structure, thus microbial uptake of ring structured salicylic acid and sinapyl alcohol was limited during the batch experiments.

There was a significant increase in the sorption of oxalic acid on sterilized Jefferson and Malmo soils (F_1,4_ = 13.1, *P* = 0.008 for Jefferson and F_1,4_ = 15.5, *P* = 0.006 for Malmo) (Fig. 3e; Table 4). Drummer soil was not considered in this comparison because precipitation of oxalate dominates over sorption in calcareous soils. The higher sorption after sterilization is most readily explained by an artifact of the γ-irradiation. We noted that the pH of soils decreased 0.2 to 0.8 units after sterilization. The order of decline was Alfisol>Ultisol>Mollisol. This pH decrease would have increased the amount of pH-dependent positive exchange sites on the mineral surfaces. This result also indicates minimal possibility of microbial uptake of oxalic acid in non-sterilized soils, otherwise the apparent sorption of oxalic acid in non-sterilized soils would have been closer to or higher than that of sterilized soils. Faster sorptive reactions than microbial reactions for carboxylic acids on soils were reported previously [66], [67].

### Conclusions

Our results showed that Ultisols exhibited approximately equal affinity to all the added compounds and Mollisols showed preferential sorption of oxalic acid and sinapyl alcohol. The sorption capacity and preference of Alfisols was in between that of Ultisols and Mollisols. This study also demonstrated significant uptake of glucose and alanine during the sorption experiment, providing clear evidence that typical sorption experiments may overestimate the sorptive capacity of soils for some sorbates. Either the sorption experiments should be conducted by minimizing the microbial activities or taking simultaneous measurements of sorption and degradation. Overall, this study provides new insights that chemical composition of DOC has a profound role in determining the extent of DOC attachment to soil solids. Detailed mechanisms of attachment of these compounds with the soil mineral and the stability of the sorbed compounds warrants further investigation. In addition, other factors contributing to the stability of OC in mineral soils such as lack of fresh OC supply to trigger microbial activities, unfavorable environmental conditions and physical hindrance between microbes and OC also needs to be addressed.
